# Draft Genome Sequence of *Clostridium botulinum* Type B Strain Osaka05, Isolated from an Infant Patient with Botulism in Japan

**DOI:** 10.1128/genomeA.01010-13

**Published:** 2014-01-23

**Authors:** Yoshihiko Sakaguchi, Koji Hosomi, Jumpei Uchiyama, Yoshitoshi Ogura, Kaoru Umeda, Masakiyo Sakaguchi, Tomoko Kohda, Masafumi Mukamoto, Naoaki Misawa, Shigenobu Matsuzaki, Tetsuya Hayashi, Shunji Kozaki

**Affiliations:** a Interdisciplinary Research Organization, University of Miyazaki, Miyazaki, Japan; b Department of Veterinary Science, Graduate School of Life and Environmental Sciences, Osaka Prefecture University, Rinku-Orai-Kita, Izumisano, Osaka, Japan; c Department of Microbiology and Infection, Faculty of Medicine, Kochi University, Kochi, Japan; d Division of Microbiology, Department of Infectious Diseases, Faculty of Medicine, University of Miyazaki, Miyazaki, Japan; e Division of Bioenvironmental Science, Frontier Science Research Center, University of Miyazaki, Miyazaki, Japan; f Department of Microbiology, Osaka City Institute of Public Health and Environmental Sciences, Tojo-cho, Tennoji-ku, Osaka, Japan; g Department of Cell Biology, Okayama University Graduate School of Medicine, Dentistry and Pharmaceutical Sciences, Kita-ku, Okayama, Japan; h Department of Veterinary Science, Faculty of Agriculture, University of Miyazaki, Gakuen Kibanadai-nishi, Miyazaki, Japan; i Graduate School of Life and Environmental Sciences, Osaka Prefecture University, Rinku-Ourai-Kita, Izumisano, Osaka, Japan

## Abstract

*Clostridium botulinum* strain Osaka05, which has been isolated from an infant patient with botulism in Japan, is the first strain producing botulinum neurotoxin subtype B6. Here, we report the draft genome sequence of *C. botulinum* Osaka05.

## GENOME ANNOUNCEMENT

*Clostridium botulinum* is a Gram-positive, spore-forming, and anaerobic bacterium. It produces botulinum neurotoxins (BoNTXs), and it causes intoxication in humans and animals (1, 2). Bacterial type and toxin type are important in *C. botulinum* because they are strongly related to the type of intoxication and host animal species. *C. botulinum* can be classified not only by culture characteristics (groups I to IV) but also by the antigenic properties of BoNTX (serotypes A to G) (3). BoNTX serotype B (BoNTX/B) can be further classified into five subtypes (B1, B2, B3, nonproteolytic B, and bivalent B), and more recently B6, due to the diversity of the amino acid sequences within serotype B (4, 5).

*C. botulinum* type B1 is generally isolated in infant and food-borne botulism. *C. botulinum* strain Osaka05, which has been isolated from an infant patient with botulism in Japan and identified as a group I strain, is the first identified *C. botulinum* producing BoNTX/B6 (5). According to pulsed-field gel electrophoresis and Southern blot analyses, the extrachromosomal DNAs have been revealed to contain the BoNTX/B6 gene in strain Osaka05 (5). Genomic investigation will extend our knowledge of *C. botulinum*. Here, we report for the first time the draft genome sequence of *C. botulinum* producing BoNTX/B6, strain Osaka05.

The DNA of strain Osaka05 was sequenced using the Roche 454 GS FLX titanium platform. A single-end library (77,710 reads) and an 8-kb paired-end library (178,004 reads) were assembled with GS Assembler software into one scaffold containing 67 gaps. Then, 22 gaps were manually closed by an *in silico* analysis using GenoFinisher software and resulted in 55 contigs (46 contigs for a chromosome and 9 contigs for extra chromosomes) (6). The draft sequence was 4,004,744 bp in total length, which is similar to the genome sizes of the other *C. botulinum* strains. The G+C content of the draft sequence (27.8%) was also similar. By use of the Microbial Genome Annotation Pipeline (MiGAP) (7), the total numbers of coding sequences (CDS) (3,671 chromosomes and 545 extrachromosomes) were predicted and at least one rRNA and at least 49 tRNAs were identified.

In the present study, the BoNTX/B6 gene was found on the Osaka05p2_contig004 (accession no. AP014517), which was considered to be a part of the extrachromosomal DNAs. Unfortunately, the complete nucleotide sequences of the extrachromosomal DNAs were not obtained in this study. Currently, it is not certain whether the extrachromosomal DNA harboring the BoNTX/B6 gene is a plasmid or bacteriophage in strain Osaka05. In the future, the complete nucleotide sequence of the extrachromosomal DNA harboring the BoNTX/B6 gene is expected to be obtained, and the evolutionary traits of the extrachromosomal DNA harboring the BoNTX/B6 gene will be analyzed.

### Nucleotide sequence accession number.

The genome sequence of *C. botulinum* strain Osaka05 was deposited in DDBJ under the accession no. BAUF00000000.

## References

[1] Cox N Hinkle R 2002 Infant botulism. Am. Fam. Physician 65:1388–1392 11996423

[2] Fox CK Keet CA Strober JB 2005 Recent advances in infant botulism. Pediatr. Neurol. 32:149–154. 10.1016/j.pediatrneurol.2004.10.001 15730893

[3] Lindström M Korkeala H 2006 Laboratory diagnostics of botulism. Clin. Microbiol. Rev. 19:298–314. 10.1128/CMR.19.2.298-314.2006 16614251 PMC1471988

[4] Hill KK Smith TJ Helma CH Ticknor LO Foley BT Svensson RT Brown JL Johnson EA Smith LA Okinaka RT Jackson PJ Marks JD 2007 Genetic diversity among botulinum neurotoxin-producing clostridial strains. J. Bacteriol. 189:818–832. 10.1128/JB.01180-06 17114256 PMC1797315

[5] Umeda K Seto Y Kohda T Mukamoto M Kozaki S 2009 Genetic characterization of *Clostridium botulinum* associated with type B infant botulism in Japan. J. Clin. Microbiol. 47:2720–2728. 10.1128/JCM.00077-09 19571018 PMC2738102

[6] Ohtsubo Y Ikeda-Ohtsubo W Nagata Y Tsuda M 2008 GenomeMatcher: a graphical user interface for DNA sequence comparison. BMC Bioinformatics 9:376. 10.1186/1471-2105-9-376 18793444 PMC2553346

[7] Sugawara H Ohyama A Mori H Kurokawa K 2009 Microbial genome annotation pipeline (MiGAP) for diverse users, abstr S-001-1-2. 20th International Conference on Genome Informatics, Yokohama, Japan

